# Cure of Ovarian Tumors by Electrolysis

**Published:** 1876-04

**Authors:** 


Cure of Ovarian Tumors by Electrolysis.—Dr. Semeleder, of Puebla, Mexico, gives (*Wiener Med. Presse*, December 26, 1875) a short account of several cases of ovarian dropsy entirely relieved by electrolysis. The rationale of the cure is explained by Dr. S. as follows: It is known that where both poles of a battery are placed in an albuminous fluid, the latter coagulates at the positive pole, while it becomes thinner at the negative. Something similar probably occurs in the ovarian cyst under the same circumstances. Not only does the fluid become absorbed, but the cyst-wall is so altered that secretion no longer takes place therefrom. This must result through the influence of the cystic fluid, for the electrodes do not come in contact with more than a very limited portion of the cyst-walls directly. It would be of the highest interest to examine, post-mortem, the condition of a cyst altered in this way and caused to shrink. Multilocular cysts are affected in a similar manner.

